# Evaluation of the Performance of Artificial Intelligence Based Chatbots in Providing First Aid Information on Dental Trauma According to the ToothSOS Application

**DOI:** 10.1111/edt.13078

**Published:** 2025-06-09

**Authors:** Ecem Elif Çege, Hamide Cömert, Neşe Akal, Ayşegül Ölmez

**Affiliations:** ^1^ Department of Paediatric Dentistry, Faculty of Dentistry Karabuk University Karabük Turkey; ^2^ Department of Paediatric Dentistry, Faculty of Dentistry Lokman Hekim University Ankara Turkey; ^3^ Department of Paediatric Dentistry, Faculty of Dentistry Gazi University Ankara Turkey

**Keywords:** artificial intelligence, ChatGPT, dental trauma, dentistry, Gemini

## Abstract

**Aim:**

The aim of this study was to evaluate the performance of ChatGPT‐4o and Gemini Advanced artificial intelligence‐based chatbots (AI‐based chatbots) in providing emergency intervention recommendations for dental trauma with intraoral photographs of patients diagnosed with traumatic dental injuries, and to assess their compatibility with emergency intervention recommendations in the ToothSOS application.

**Material and Methods:**

In this study, 80 intraoral photographs obtained from patients presenting with dental trauma were uploaded to two different AI‐based chatbots (ChatGPT‐4o and Gemini Advanced) and the responses generated by these systems were evaluated by four paediatric dentists. The evaluators scored the responses with a Modified Global Quality Score (GQS), referring to the English instructions of the ToothSOS application. In order to analyse the reliability of the responses, a total of three evaluation sessions were conducted 1 week apart.

**Results:**

The ChatGPT‐4o performed better when all injury types were considered together (*p* = 0.012). It was found that the ChatGPT‐4o performed much better in complicated crown fracture cases (*p* = 0.004) and the Gemini Advanced chatbot performed much better in critical dental injuries such as avulsion (*p* < 0.001).

**Conclusions:**

AI‐based chatbots can be a helpful tool in the assessment of dental trauma. However, further development and expert validation are needed to improve their accuracy and consistency, especially in complex cases. Incorporating the International Association of Dental Traumatology (IADT) guidelines into the databases of these systems could improve the reliability of their recommendations. In addition, given the widespread use of AI‐based chatbots in many fields, particularly health, they could contribute to public health by supporting access to accurate information.

## 1 Introduction

Traumatic dental injuries (TDI's) are common among children and young adults, accounting for 5% of all injuries [1]. A meta‐analysis reported that one billion people worldwide have experienced a TDI [2]. TDIs are considered emergency situations requiring immediate intervention [3]. It is crucial for the general public to be aware of first aid measures for TDIs; however, existing studies indicate that knowledge in this area is insufficient [4, 5].

With recent technological advances, people can access health information through online platforms (e.g., mobile applications, chatbots) and the use of AI‐based chatbots as a source of healthcare information has increased. Chatbots can provide information about treatment, side effects, prognosis, and outcomes by answering questions asked by the public [6, 7]. Studies have shown that 42%–71% of adults who use the internet for health purposes primarily do so to access information [8, 9]. For these reasons, applications and AI‐based chatbots are considered to have the potential to be highly beneficial in dental trauma cases, where timely intervention is crucial.

**FIGURE 1:**
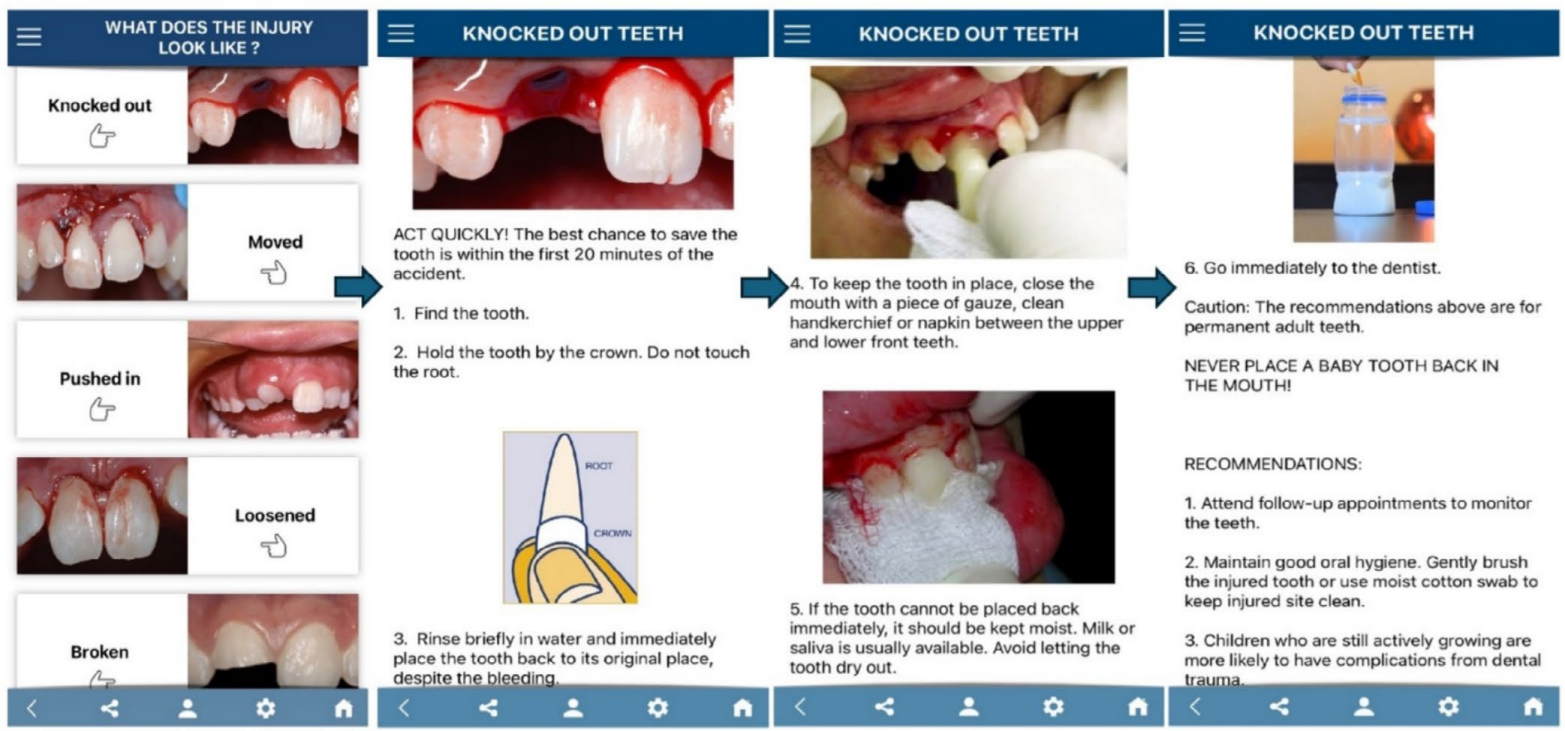
Patient section of the ToothSOS app, types of dental injuries and advice screens.

The ToothSOS application, designed and developed by the IADT Education Committee and validated by an international panel of experts selected by the IADT, has aimed to provide the general public with information on dental trauma since April 2018. ToothSOS is available as a free download for both Apple and Android devices. The “*Patient*” section provides first‐aid guidance for dental trauma before professional care. It includes step‐by‐step instructions for managing fractures, displacements, impactions, avulsions, and injuries affecting the skin, lips, gums, jaws, and joints (Figure 1) [10]. All trauma management protocols follow the official IADT guidelines [11].

Large Language Models (LLMs) are systems that aim to simulate human speech using artificial intelligence (AI) methods. LLM‐based chatbots help users understand complex topics more easily by presenting information in a conversational format. LLMs process only text‐based inputs to generate language and perform linguistic analysis, whereas Large Multimodal Models (LMMs), such as GPT‐4 [12], PaLM [13], and Gemini [14], can process multiple data modalities, including text, images, audio, and video. The first AI chatbot is ChatGPT (Chat Generative Pre‐Trained Transformer), which was announced by OpenAI Inc. (San Francisco, CA, USA) on November 30, 2022. Within the first 3 months following its release, it broke records by reaching 100 million new users [15]. ChatGPT‐4, which supports image inputs and is based on GPT‐4 Turbo, was introduced in March 2023 [16]. The latest and faster version, ChatGPT‐4o, was released in May 2024.

Another AI chatbot with the capability to process image inputs is Gemini Advanced (Google DeepMind, Google LLC, Mountain View, CA, USA) and is based on Gemini 1.5 Pro [14]. LMMs have demonstrated strong performance in encoding clinical knowledge and performing medical question‐answering tasks [14, 17, 18, 19]. Additionally, users who are not health professionals are increasingly relying on AI‐based chatbots as a source of health information [20].

In the existing literature, there are studies on the performance of AI‐based chatbots in answering dentistry‐ and TDI‐related questions [21, 22, 23, 24, 25]. However, no study has been found that utilizes image‐based inputs.

The aim of this study was to evaluate the performance of ChatGPT‐4o and Gemini Advanced AI‐based chatbots in providing emergency intervention recommendations for dental trauma with intraoral photographs of patients diagnosed with TDI, and to assess their compatibility with emergency intervention recommendations in the ToothSOS application. At the same time, it will determine whether there is a difference between the knowledge levels of the two AI‐based chatbots. No study has yet been identified in the literature that compares the data from the ToothSOS application with the answers given by the AI‐based chatbots.

## 2 Material and Methods

### 2.1 Data Collection

Intraoral photographs, trauma types, and treatments of patients admitted to …. University Department of Pediatric Dentistry due to dental trauma are routinely documented in the archive with informed consent. For this study, 80 intraoral photographs of patients treated between January 2020 and December 2024 were retrieved from the department archive.

### 2.2 Sample Size

In this study, the sample size was calculated at a 95% confidence level using the “G. Power‐3.1.9.2” software. The required sample size for testing the research hypothesis was determined at an *α* = 0.05 significance level, with a theoretical power of 0.95, using a standardized effect size of 0.638 obtained from a previous similar study [25]. As a result of the analysis, the minimum required sample size was calculated to be 36.

### 2.3 Data Evaluation

In this study, a total of 87 intraoral photographs were selected from the 106 images available in the clinical archive. To ensure standardization and focus on isolated dental trauma cases, photographs that included soft tissue injuries, bone fractures, or complex trauma involving multiple trauma types were excluded.

The intraoral image of each selected case was saved in a standardized format (maximum resolution: 1920 × 1080 pixels, file format: JPG). Access to the ChatGPT‐4o and Gemini Advanced was provided via Google search engine. To minimize algorithmic and contextual bias, browser history and cookies were cleared before each question‐and‐answer session. A new email address was used for every session to eliminate personalized response effects. While the same account was used within a given session, a new chat window was opened for each case to avoid any influence from previous prompts. The intraoral photographs were presented to two different AI‐ based chatbots (ChatGPT‐4o and Gemini Advanced) by a dentist who was not involved in the evaluation process. For each case, both chatbots were asked the same question under identical conditions: “My tooth hurts. You can see it in the photo. What should I do before seeing a dentist?” When a case image was uploaded to the chat, this question was entered, and the responses were recorded in writing. The responses were randomly labeled as A or B, and the correspondence between the responses and the chatbots was stored separately. The recorded responses were then evaluated by four pediatric dentists.

Each evaluator used the ToothSOS app's English‐language instructions (Figure 1) as the “gold standard” for comparison. The responses were rated using the Modified Global Quality Score (GQS) based on a 5‐point Likert scale (Table 1) [26].

**TABLE 1:** Modified Global Quality Scale (GQS) [26].

Score	Description
Score 5 (Strongly Agree)	The response is accurate, and the content is comprehensive
Score 4 (Agree)	The response is accurate, and most of the content is correct, but there are some omissions or inaccuracies
Score 3 (Neutral)	The response is partially correct, but the details are largely incorrect, incomplete, or irrelevant
Score 2 (Disagree)	The response is incorrect, but some correct elements are present
Score 1 (Strongly Disagree)	The response and all content are completely incorrect or irrelevant

One week and 2 weeks after the first question answering system, a total of three times, the question answering system was repeated on the same day. Since the “gold standard” was met with GQS, no further calibration was required.

**FIGURE 2:**
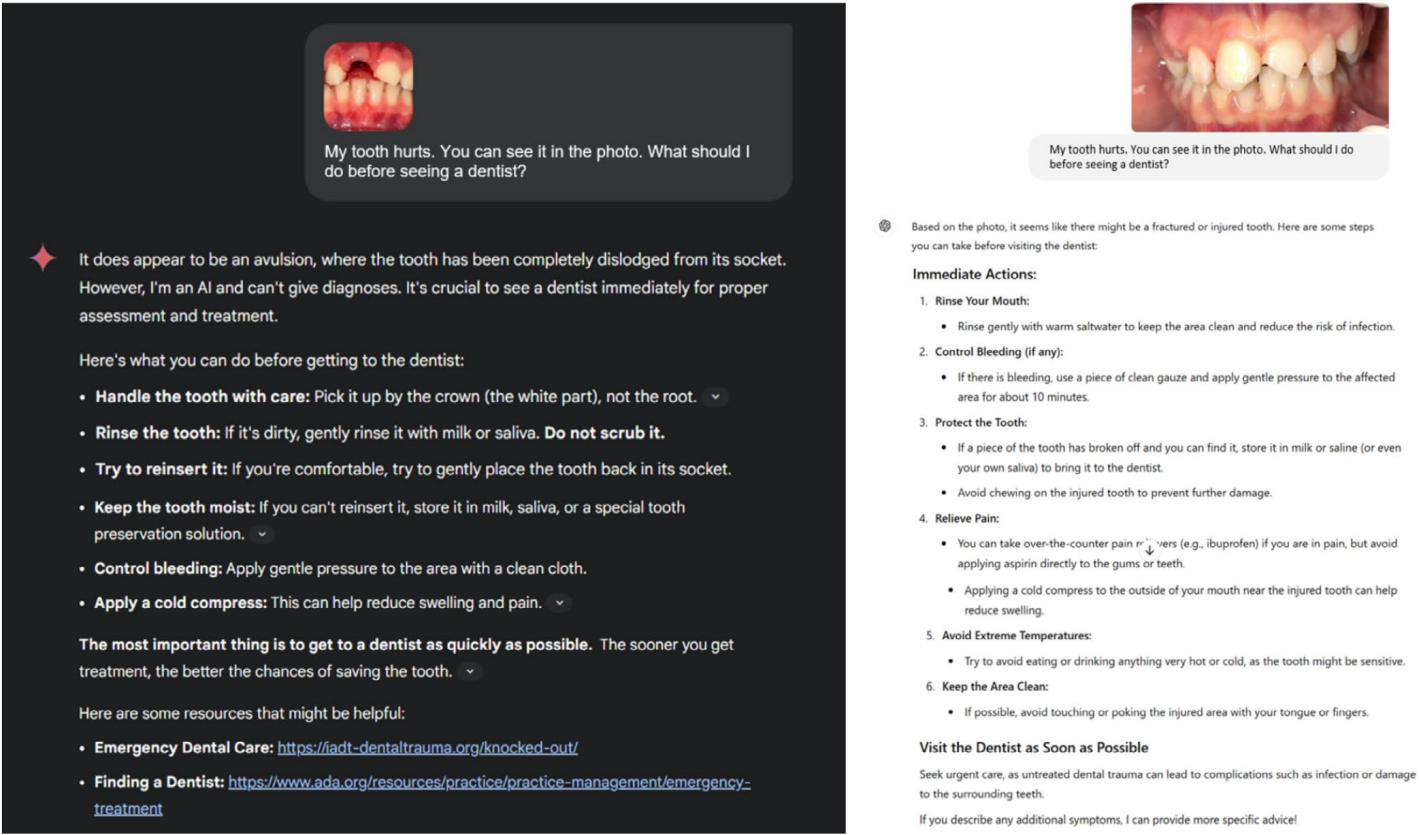
Representative high‐quality responses from two different AI‐based chatbots to intraoral photographs related to dental trauma cases.

**FIGURE 3:**
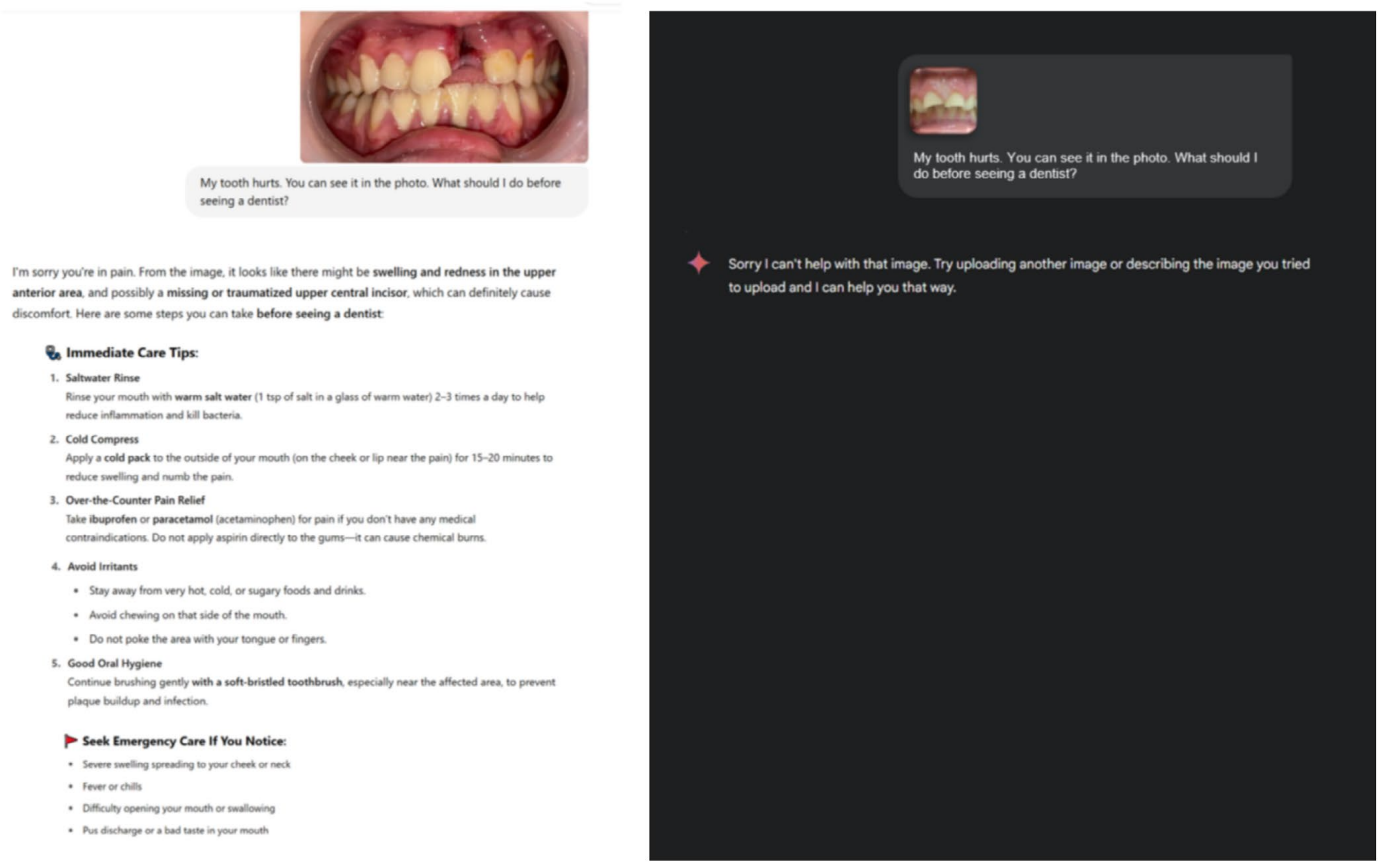
Representative low‐quality responses from two different AI‐based chatbots to intraoral photographs related to dental trauma cases.

When evaluating Gemini Advanced's response to patient images, it was observed that the ModelAI‐based chatbot was unable to process certain intraoral photographs. Specifically, when an evaluator uploaded images of a tooth injury, the model responded with “Sorry, I can't help with that image. Try uploading another image or describing the image you tried to upload, and I can help you that way.” Due to this limitation, 7 of the 87 images (cases 2, 23, 28, 65, 67, 77, and 78) were excluded from the study and the study was completed with 80 intraoral images. This limitation is thought to stem from Gemini Advanced's privacy and security policies. For this reason, the responses of two different AI‐based chatbots to 80 intraoral photographs in three different time periods were evaluated by four pediatric dentists using the GQS. To illustrate the evaluation process, examples of responses provided by the two different AI‐based chatbots to the intraoral photographs are presented in Figures 2 and 3.

Of the intraoral photographs included in the study, 22 were uncomplicated crown fractures, 27 were complicated crown fractures, 9 were avulsions, 9 were intrusions, and 13 were luxation injuries. Only one type of TDI was present in each photograph.

### 2.4 Statistical Analysis

In this study, intraclass correlation and kappa test–retest tests were used for categorical variables to test whether repeated values of the same variable were similar. Fisher's exact test was used to test the relationship between categorical variables when the sample size assumption (expected value > 5) was not met. The marginal homogeneity test was used to test the relationship between dependent categorical variables. Analyses were performed using IBM SPSS 27 software.

## 3 Results

In this study, the compatibility of the performance of ChatGPT‐4o and Gemini Advanced AI‐based chatbots in providing first aid information for dental trauma with the emergency intervention recommendations in the ToothSOS application was investigated.

The agreement between the assessments made by four independent pediatric dentists was analyzed using the Kappa coefficient, and the coefficient values ranged from 0.634 to 0.663 (Table 2). These values indicate a moderate to high level of agreement among the evaluators. For all Kappa coefficients, *p* < 0.001, indicating that the observed agreement was statistically significant.

**TABLE 2:** Intraclass Correlation Coefficient (ICC) and inter‐rater agreement (Kappa).

Evaluators	ICC	*p*	Kappa	*p*
Observer 1	0.756	< 0.001*	Day 1	0.663
Observer 2	0.792	< 0.001*	Day 2	0.642
Observer 3	0.776	< 0.001*	Day 3	0.634
Observer 4	0.754	< 0.001*	—	—

*Note: p* < 0.05.

The reliability of assessments at different measurement times was evaluated using the Intraclass Correlation Coefficient (ICC), with calculated ICC values ranging from 0.754 to 0.792 (Table 2). All ICC values exceeded 0.700, indicating a high level of reliability between the assessments. Moreover, *p* < 0.001, confirming that this agreement was statistically significant.

### 3.1 Analysis of Responses According to Artificial Intelligence Models

There were statistically significant differences between the AI‐based chatbots when overall responses were evaluated (*p* = 0.012). ChatGPT‐4o showed higher overall positive response rates (“Agree” and “Strongly Agree”) (71.3%), while Gemini Advanced showed a more balanced and more diverse response distribution (Table 3).

**TABLE 3:** Distribution of responses by AI‐based chatbots for injury types.

Injury type	AI‐based chatbots	Strongly Disagree % (*n*)	Disagree % (*n*)	Neutral % (*n*)	Agree % (*n*)	Strongly Agree % (*n*)	Test statistic	*p*
Uncomplicated crown fracture	ChatGPT	0.0 (0)	4.2 (1)	8.3 (2)	25.0 (6)	62.5 (15)	5.670	0.132
	Gemini	0.0 (0)	16.7 (4)	25.0 (6)	25.0 (6)	33.3 (8)		
Complicated crown fracture	ChatGPT	0.0 (0)	3.6 (1)	0.0 (0)	32.1 (9)	64.3 (18)	13.032	0.004*
	Gemini	10.7 (3)	10.7 (3)	17.9 (5)	35.7 (10)	25.0 (7)		
Avulsion	ChatGPT	10.0 (1)	70.0 (7)	20.0 (2)	0.0 (0)	0.0 (0)	13.581	< 0.001*
	Gemini	40.0 (4)	0.0 (0)	10.0 (1)	20.0 (2)	30.0 (3)		
Luxation	ChatGPT	0.0 (0)	21.4 (3)	28.6 (4)	50.0 (7)	0.0 (0)	1.418	1.000
	Gemini	7.1 (1)	28.6 (4)	21.4 (3)	42.9 (6)	0.0 (0)		
Intrusion	ChatGPT	0.0 (0)	25.0 (1)	25.0 (1)	50.0 (2)	0.0 (0)	1.767	1.000
	Gemini	25.0 (1)	25.0 (1)	25.0 (1)	25.0 (1)	0.0 (0)		
Total	ChatGPT	1.3 (1)	16.3 (13)	11.3 (9)	30.0 (24)	41.3 (33)	12.832	0.012*
	Gemini	11.3 (9)	15.0 (12)	20.0 (16)	31.3 (25)	22.5 (18)		

* *p* < 0.05.

**FIGURE 4:**
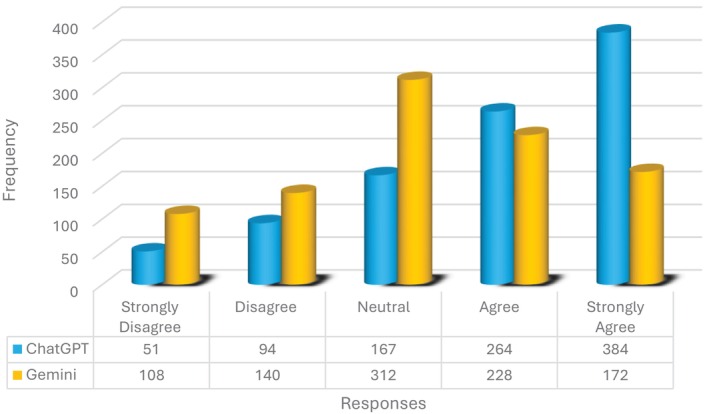
Distribution of all responses provided by AI‐based chatbots based on evaluations by evaluators across three separate assessment sessions.

The distribution of responses provided by AI‐based chatbots for different TDI types is presented in Figure 4, and Fisher's Exact tests were conducted to analyze the relationships between them. The analyses revealed statistically significant differences among AI‐based chatbots for complicated crown fractures (*p* = 0.004) and avulsion (*p* < 0.001) (Tables 3 and 4). For complicated crown fractures, responses from ChatGPT‐4o were predominantly positive (32.1% “Agree” and 64.3% “Strongly Agree”), whereas Gemini Advanced showed a more heterogeneous distribution (*p* = 0.004).

**TABLE 4:** Distribution of responses by injury type for AI‐based chatbots.

	Uncomplicated crown fracture % (*n*)	Complicated crown fracture % (*n*)	Avulsion % (*n*)	Luxation % (*n*)	Intrusion % (*n*)	Test statistic	*p*
ChatGPT							
Strongly Disagree	0.0 (0)	0.0 (0)	10.0 (1)	0.0 (0)	0.0 (0)	58.877	< 0.001*
Disagree	4.2 (1)	3.6 (1)	70.0 (7)	21.4 (3)	25.0 (1)		
Neutral	8.3 (2)	0.0 (0)	20.0 (2)	28.6 (4)	25.0 (1)		
Agree	25.0 (6)	32.1 (9)	0.0 (0)	50.0 (7)	50.0 (2)		
Strongly Agree	62.5 (15)	64.3 (18)	0.0 (0)	0.0 (0)	0.0 (0)		
Gemini							
Strongly Disagree	0.0 (0)	10.7 (3)	40.0 (4)	7.1 (1)	25.0 (1)	21.792	0.072
Disagree	16.7 (4)	10.7 (3)	0.0 (0)	28.6 (4)	25.0 (1)		
Neutral	25.0 (6)	17.9 (5)	10.0 (1)	21.4 (3)	25.0 (1)		
Agree	25.0 (6)	35.7 (10)	20.0 (2)	42.9 (6)	25.0 (1)		
Strongly Agree	33.3 (8)	25.0 (7)	30.0 (3)	0.0 (0)	0.0 (0)		
Total							
Strongly Disagree	0.0 (0)	5.4 (3)	25.0 (5)	3.6 (1)	12.5 (1)	56.922	< 0.001*
Disagree	10.4 (5)	7.1 (4)	35.0 (7)	25.0 (7)	25.0 (2)		
Neutral	16.7 (8)	8.9 (5)	15.0 (3)	25.0 (7)	25.0 (2)		
Agree	25.0 (12)	33.9 (19)	10.0 (2)	46.4 (13)	37.5 (3)		
Strongly Agree	47.9 (23)	44.6 (25)	15.0 (3)	0.0 (0)	0.0 (0)		

* *p* < 0.05.

Evaluators mostly provided positive responses (“Agree” and “Strongly Agree”) for all crown fractures when assessing ChatGPT‐4o's performance, but they tended to respond negatively (“Disagree”) for avulsion and intrusion injuries (Tables 3, 4). Gemini Advanced received a more balanced distribution of responses from the evaluators, except for avulsion injuries. Statistical analyses highlighted significant differences between the models, particularly for avulsion cases (*p* < 0.001). Evaluators rated ChatGPT‐4o with “Disagree” (70%) and “Neutral” (20%) responses, whereas Gemini Advanced received a higher proportion of “Agree” (20%) and “Strongly Agree” (30%) responses, aligning more closely with ToothSOS recommendations.

### 3.2 Analysis of AI‐Based Chatbot Response Changes Over Time

The distribution of all responses provided by AI‐based chatbots at different evaluation times was analyzed using marginal proportion tests to evaluate changes over time. A statistically significant difference was found between the responses given by ChatGPT‐4o in the first and second weeks (*p* < 0.05) (Table 5). It was observed that responses changed in the second week, with an increase in the proportion of “Agree” and “Strongly Agree” responses. In contrast, no significant change was observed in Gemini Advanced responses over time (*p* > 0.05) (Table 6).

**TABLE 5:** Distribution of responses by evaluation times for ChatGPT.

Second week	Strongly Disagree % (*n*)	Disagree % (*n*)	Neutral % (*n*)	Agree % (*n*)	Strongly Agree % (*n*)	Test statistic	*p*
First week							
Strongly Disagree	0.0 (0)	40.0 (4)	0.0 (0)	0.0 (0)	0.0 (0)	−2495	0.013*
Disagree	0.0 (0)	20.0 (2)	9.1 (1)	4.0 (1)	2.9 (1)		
Neutral	0.0 (0)	20.0 (2)	63.6 (7)	24.0 (6)	14.7 (5)		
Agree	0.0 (0)	10.0 (1)	18.2 (2)	48.0 (12)	41.2 (14)		
Strongly Agree	0.0 (0)	10.0 (1)	9.1 (1)	24.0 (6)	41.2 (14)		
Third week							
Strongly Disagree	0.0 (0)	10.0 (1)	0.0 (0)	0.0 (0)	0.0 (0)	−0.943	0.346
Disagree	0.0 (0)	60.0 (6)	9.1 (1)	16.0 (4)	5.9 (2)		
Neutral	0.0 (0)	20.0 (2)	36.4 (4)	12.0 (3)	0.0 (0)		
Agree	0.0 (0)	10.0 (1)	45.5 (5)	36.0 (9)	26.5 (9)		
Strongly Agree	0.0 (0)	0.0 (0)	9.1 (1)	36.0 (9)	67.6 (23)		

Third week	Strongly Disagree % (*n*)	Disagree % (*n*)	Neutral % (*n*)	Agree % (*n*)	Strongly Agree % (*n*)	Test statistic	*p*
First week							
Strongly Disagree	100.0 (1)	0.0 (0)	0.0 (0)	0.0 (0)	0.0 (0)	−1688	0.091
Disagree	0.0 (0)	100.0 (13)	0.0 (0)	0.0 (0)	0.0 (0)		
Neutral	0.0 (0)	0.0 (0)	100.0 (9)	0.0 (0)	0.0 (0)		
Agree	0.0 (0)	0.0 (0)	0.0 (0)	100.0 (24)	0.0 (0)		
Strongly Agree	0.0 (0)	0.0 (0)	0.0 (0)	0.0 (0)	100.0 (33)		

* *p* < 0.05.

**TABLE 6:** Distribution of responses by evaluation times for Gemini.

Second week	Strongly Disagree % (*n*)	Disagree % (*n*)	Neutral % (*n*)	Agree % (*n*)	Strongly Agree % (*n*)	Test statistic	*p*
First week							
Strongly Disagree	70.0 (7)	0.0 (0)	0.0 (0)	0.0 (0)	0.0 (0)	0.901	0.368
Disagree	0.0 (0)	14.3 (1)	3.7 (1)	13.0 (3)	7.7 (1)		
Neutral	10.0 (1)	28.6 (2)	40.7 (11)	39.1 (9)	30.8 (4)		
Agree	0.0 (0)	28.6 (2)	37.0 (10)	39.1 (9)	46.2 (6)		
Strongly Agree	20.0 (2)	28.6 (2)	18.5 (5)	8.7 (2)	15.4 (2)		
Third week							
Strongly Disagree	80.0 (8)	0.0 (0)	0.0 (0)	0.0 (0)	7.7 (1)	−0.747	0.455
Disagree	10.0 (1)	28.6 (2)	7.4 (2)	17.4 (4)	23.1 (3)		
Neutral	0.0 (0)	28.6 (2)	29.6 (8)	21.7 (5)	7.7 (1)		
Agree	0.0 (0)	42.9 (3)	37.0 (10)	43.5 (10)	15.4 (2)		
Strongly Agree	10.0 (1)	0.0 (0)	25.9 (7)	17.4 (4)	46.2 (6)		

Third week	Strongly Disagree % (*n*)	Disagree % (*n*)	Neutral % (*n*)	Agree % (*n*)	Strongly Agree % (*n*)	Test statistic	*p*
First week							
Strongly Disagree	100.0 (9)	0.0 (0)	0.0 (0)	0.0 (0)	0.0 (0)	0.18	0.861
Disagree	0.0 (0)	100.0 (12)	0.0 (0)	0.0 (0)	0.0 (0)		
Neutral	0.0 (0)	0.0 (0)	100.0 (16)	0.0 (0)	0.0 (0)		
Agree	0.0 (0)	0.0 (0)	0.0 (0)	100.0 (25)	0.0 (0)		
Strongly Agree	0.0 (0)	0.0 (0)	0.0 (0)	0.0 (0)	100.0 (18)		

## 4 Discussion

In this study, the performance of ChatGPT‐4o and Gemini Advanced AI‐based chatbots in providing first aid information for dental trauma is examined in terms of their compatibility with the emergency response recommendations in the ToothSOS application. In this context, the performance of the two chatbots was analyzed by the evaluators in a total of three evaluations on different days. High reliability was found in terms of interobserver agreement and consistency over time (*p* < 0.001).

Prior to the present study, no studies in the field of dentistry with visual input to AI‐based chatbots were found in the literature. Existing studies in dentistry have generally been conducted with text‐based input [21, 25, 27, 28]. Some of these studies used ChatGPT‐3.5, which does not accept image input and is a free subversion [21, 25, 27].

In the study by Öztürk et al. on TDIs, ChatGPT 3.5 and ChatGPT 4.0 were evaluated in terms of responses to questions related to TDI for dental students and professionals, and it was found that ChatGPT 4.0 had a statistically higher GQS than ChatGPT 3.5 in the definition and diagnosis section [27]. In a study comparing ChatGPT 3.5 and Gemini and assessing the accuracy and comprehensiveness of responses to 33 text‐based questions on dental avulsion only, Gemini responses were statistically significantly more accurate than ChatGPT (*p* = 0.004) [25]. In another study, ChatGPT 3.5, ChatGPT‐4o, and Google Gemini were asked 59 text‐based TDI questions taken from popular question‐answer sites or manually generated based on hypothetical case scenarios. As a result, ChatGPT‐4o and Google Gemini were rated significantly higher in terms of quality compared to ChatGPT 3.5 [21]. In a text‐based study with multiple‐choice questions prepared from undergraduate endodontic education topics, ChatGPT‐4 and ChatGPT‐4o were compared and the accuracy rate of ChatGPT‐4o (92.8%) was significantly higher than that of ChatGPT‐4 (81.7%; *p* < 0.001) [28]. For these reasons, ChatGPT‐4o and Gemini Advanced that accept image input were used in the current study.

When all responses are evaluated together, it is seen that ChatGPT‐4o's responses are rated as “Agree” (30.0%) and “Strongly Agree” (41.3%) more than Gemini Advanced. In the study conducted by Mustuloğlu and Deniz, in which text‐based true/false questions prepared by paediatric dentists were submitted to six different AI‐based chatbots at different times, ChatGPT 4.0 was reported to achieve the highest accuracy rate at 95.6% across all time periods [23]. Similarly, in the study conducted by Salem et al. in which they aimed to evaluate the performance of 4 chatbots in answering multiple‐choice questions in endodontics, it was found that ChatGPT‐4o performed better than Gemini Advanced [29].

In the existing literature, there is no AI‐based chatbot study in which different TDI types are considered together. In this study, significant differences were found between ChatGPT‐4o and Gemini Advanced in avulsion and complicated crown fracture cases. Although ChatGPT‐4o provided more favorable responses in complicated and uncomplicated crown fractures, it was inadequate in avulsion cases. Gemini Advanced showed greater alignment with ToothSOS recommendations. These performance differences may stem from the structural tendencies of the models. ChatGPT‐4o appears to perform better in contextual and descriptive tasks, such as crown fractures, while Gemini tends to follow stepwise emergency protocols more rigidly—making it more reliable in cases like avulsion. This highlights the need to consider model‐specific behavior when interpreting AI performance across trauma types. This finding is similar to the results of another study conducted with text‐based questions about dental avulsion, in which the accuracy and comprehensiveness of the answers given by ChatGPT 3.5 and Gemini were evaluated using GQS and it was found that Gemini's answers about dental avulsion were more accurate than ChatGPT (*p* = 0.004) [25]. However, the AI‐based chatbots used in this study are a sub‐version of the models used in the current study. Contrary to the results of our study, in a similar study by Mustuloglu et al. in which 18 true/false questions related to dental avulsion were asked, it was stated that AI‐based chatbots, except for ChatGPT‐4o, were not yet sufficiently reliable sources for use in emergency management of dental avulsion [23].

In this study, measures such as clearing search history and cookies were taken at each question‐answer stage to avoid bias, as in a similar study [25]. However, when the findings were analysed, a significant change was observed in ChatGPT‐4o performance between the first and second weeks (*p* < 0.05). On the other hand, it was determined that the responses of Gemini Advanced in three different time periods were more consistent according to the injury types, and these differences were not statistically significant (*p* > 0.05). In contrast, the observed variation in ChatGPT‐4o's responses over time may be related to ongoing model updates, backend changes, or its non‐deterministic nature. While such changes may improve the model's knowledge base, they also raise concerns about reproducibility and consistency in real‐world emergency contexts. This aligns with our finding that ChatGPT‐4o's responses in the second evaluation were more accurate compared to the first. Future studies should therefore record the model version and date of use to ensure transparency and comparability. In the study of Özden et al., the same dental trauma questions were asked to ChatGPT 3.5 and Google Bard (Gemini) for 10 days, and the consistency and accuracy of the answers were analyzed. Similar to our study, Google Bard was found to give more consistent answers, but both applications did not reach a sufficient level of consistency for clinical use [24]. It should be taken into account that the responses may vary in evaluations conducted at different times, as AI‐based chatbots are regularly updated by their development teams and adapted based on user feedback.

One of the limitations of this study is that chatbot responses for emergency situations were expected to be brief; therefore, readability metrics such as Flesch–Kincaid Grade Level (FKGL) scores were not assessed. Instead, response quality was evaluated solely using the Global Quality Scale (GQS), with the ToothSOS application serving as the reference standard. Although the researchers did not observe responses containing overly technical or medical terminology, the readability and comprehensibility of chatbot outputs for the general public were not systematically evaluated. Future studies are recommended to include readability assessments to better assess public accessibility and user‐friendliness.

Another limitation of the study is the high quality of intraoral photographs obtained from the clinical archive, as they were captured under ideal clinical conditions using retractors and mouth mirrors. However, in real‐life emergency scenarios, images are often submitted by parents using smartphones under suboptimal conditions—such as poor lighting or improper positioning—which may result in blurry, low‐resolution, or incomplete photographs. This limits the generalizability of the findings to real‐life situations. Additionally, soft tissue injuries were excluded from analysis since images containing blood and similar elements could not be processed due to the privacy and security policies of chatbots, particularly Gemini Advanced. Furthermore, the number of intraoral photographs was not evenly distributed among different types of TDIs. The study included 22 uncomplicated crown fractures, 27 complicated crown fractures, 9 avulsions, 9 intrusions, and 13 dislocations. Given that crown fractures are more common than severe TDIs such as avulsions, it is likely that more images of crown fractures were available in the clinical archive. A notable limitation is that the ChatGPT‐4o used in this study is a paid version, restricting access to all potential users.

While this study provides an initial benchmark for evaluating AI chatbot performance using image‐based dental trauma scenarios, we acknowledge that our analytical approach has limitations. Due to the categorical nature of the data and the relatively small number of clinical image cases, we employed descriptive and non‐parametric statistical methods suitable for early‐stage evaluation. However, future studies with larger datasets and standardized, structured outputs could benefit from more advanced techniques—such as multivariate analysis or machine learning‐based modeling—to explore interaction effects across trauma types, chatbot versions, and time points. Incorporating these methods may enhance the understanding of AI behavior in dynamic clinical scenarios and contribute to more robust performance evaluation frameworks.

As a result:

- The ChatGPT‐4o performed better when all injury types were considered together (*p* = 0.012).
- Statistically significant differences were observed between the recommendations given by the AI‐based chatbots in complicated crown fracture and avulsion cases (*p* < 0.05). It was found that the ChatGPT‐4o performed significantly better in complicated crown fracture cases, while Gemini Advanced performed much better in critical dental injuries such as avulsion.
- In other injury types, AI‐based chatbots showed similar performance and the difference between them was not statistically significant (*p* > 0.05).

This study evaluates the reliability and accuracy of the recommendations of AI‐based chatbots for dental trauma and demonstrates the potential for clinical use of AI against different types of trauma. Incorporating the IADT guidelines into the databases of AI‐based chatbots can improve the accuracy and consistency of these robots. In addition, given the increasing use of AI‐based chatbots in accessing all kinds of information, especially health, it can contribute to the access of societies to accurate information.

## Author Contributions

Conception, data collection, statistical analysis: E.E.Ç., H.C., N.A., and A.Ö. Manuscript writing: E.E.Ç. and H.C. Review and editing the manuscript: N.A. and A.Ö. All authors read and approved the final manuscript.

## Ethics Statement

The study was approved by the Gazi University Ethics Committee on 14.01.2025 with the decision number E‐77082166‐604.01‐1143617.

## Consent

Each patient signed an informed consent form.

## Conflicts of Interest

The authors declare no conflicts of interest.

## Data Availability

The data that support the findings of this study are available from the corresponding author upon reasonable request.
